# Immersive Low-Cost Virtual Reality Treatment for Phantom Limb Pain: Evidence from Two Cases

**DOI:** 10.3389/fneur.2018.00067

**Published:** 2018-02-19

**Authors:** Elisabetta Ambron, Alexander Miller, Katherine J. Kuchenbecker, Laurel J. Buxbaum, H. Branch Coslett

**Affiliations:** ^1^Laboratory for Cognition and Neural Stimulation, Department of Neurology, Perelman School of Medicine at the University of Pennsylvania, Philadelphia, PA, United States; ^2^Haptic Intelligence Department, Max Planck Institute for Intelligent Systems, Stuttgart, Germany; ^3^Cognition and Action Laboratory, Moss Rehabilitation Research Institute, Philadelphia, PA, United States

**Keywords:** phantom limb, phantom limb pain, amputee, virtual reality, mirror box

## Abstract

Up to 90% of amputees experience sensations in their phantom limb, often including strong, persistent phantom limb pain (PLP). Standard treatments do not provide relief for the majority of people who experience PLP, but virtual reality (VR) has shown promise. This study provides additional evidence that game-like training with low-cost immersive VR activities can reduce PLP in lower-limb amputees. The user of our system views a real-time rendering of two intact legs in a head-mounted display while playing a set of custom games. The movements of both virtual extremities are controlled by measurements from inertial sensors mounted on the intact and residual limbs. Two individuals with unilateral transtibial amputation underwent multiple sessions of the VR treatment over several weeks. Both participants experienced a significant reduction of pain immediately after each VR session, and their pre-session pain levels also decreased greatly over the course of the study. Although preliminary, these data support the idea that VR interventions like ours may be an effective low-cost treatment of PLP in lower-limb amputees.

## Introduction

Individuals who undergo amputation commonly experience the sensation that the missing extremity is still present, a phenomenon known as a “phantom limb” (PL) (1). A significant proportion of individuals who experience a PL—from 65 to 70% in many studies—also experience persistent and debilitating pain in the missing limb, a condition known as phantom limb pain (PLP) (2, 3). PLP typically appears immediately after or within 1 week of amputation, but in rare cases it has been reported to begin months or years after amputation (1). Its frequency and characteristics vary across individuals. PLP can be sporadic or steady, and it can be experienced as burning, tingling, throbbing, cramping, squeezing, shocking, or shooting (4). Furthermore, some individuals may also report foreshortening of the PL, a phenomenon known as “telescoping,” which is associated with an increase in PLP (5, 6).

Although the cause of PLP is unclear, a number of hypotheses regarding the etiology of the disorder have been advanced. Some accounts attribute the deficit to peripheral nervous system disorders such as neuromas (5, 7). The transection of the nerve with the limb amputation and the consequent development of neuromas can induce ectopic discharges and the sensation of pain. The fact that anesthetic blockade of the nerve reduces pain in some amputees (8) indicates that this explanation accounts for PLP in some instances. However, not all individuals experience a reduction in PLP from the use of anesthetic at the residual limb (9). This observation, in addition to the occurrence of PLP in individuals with congenital absence of an extremity (10, 11), suggests that the disorder arises from more central alterations.

It has been proposed that the amputation of a limb may induce a “cortical remapping” at the level of somatatosensory and motor cortices. Animal studies have shown that amputation of a limb induces neighboring areas to invade the cortical regions that represent the amputated body part (5, 12, 13). This interpretation has been supported with behavioral and neuroimaging evidence in humans, which showed that tactile stimulation of the face (represented cortically in close proximity to the hand area), but not of other parts of the body, is perceived as stimulation of the PL and induces an activation of the hand area (14). This cortical remapping of somatosensory as well as motor cortex has been proposed as one of the possible mechanisms responsible for PLP (15, 16). Flor and colleagues (17, 18) showed that PLP, but not PL phenomena *per se*, correlated with the level of cortical remapping. A possible mechanism for this cortical remapping is the “noise” produced by neuromas or the loss of C-fibers after amputation (5).

An alternative account links the cortical remapping interpretation with the observation that individuals who experience PLP often noted pain before the amputation (19). This theory proposes the existence of some memory for pain mechanisms (5). The long-lasting activation of nociceptors prior to amputation of the limb may induce alterations at the level of primary sensory cortex (5) or at multiple sites in the “pain matrix” (4). With limb amputation and consequent cortical remapping, expansion of the neighboring areas into the cortical area of the amputated limb might induce reactivation of the memory for pain that is coded in these regions and elicit the experience of PLP (5). While this interpretation can account for PLP in some individuals who experience chronic pain (19), it cannot explain PLP in individuals with amputation from trauma.

Yet another account attributes PLP to a disruption of the primary sensory–motor representation of the missing extremity, a phenomenon sometimes called “maladaptive plasticity” (5, 20). This interpretation rests on the fact that the ability to generate motor commands remains intact after the amputation. Indeed, studies have documented preserved activation of motor areas in individuals who experience a PL (21), as though the limb were still present (22). The motor commands sent to an amputated limb, however, fail to generate the visual, auditory, proprioceptive, and tactile afferent signals that the brain expects (1, 23). The lack of correspondence between action plans and sensory feedback from action is hypothesized to introduce imprecision, or “noise,” in the representation of the extremity, and this imprecision may manifest as pain. A variant of this account has been suggested by recent evidence from Makin and colleagues [e.g., Ref. (24, 25)] that the integrity of hand cortical representations (and disconnection of these intact representations from sensory input) is associated with PL or PLP phenomenon. Finally, mood, anxiety, and other psychological factors also play a role in PLP (5, 7).

These varied explanations for PLP are not mutually exclusive and may together account for the observed differences in PLP across individuals (6). The variability in PLP etiology and characteristics may also explain why certain individuals respond more or less well to particular treatments (26). Indeed, several different therapies have demonstrated benefit in some individuals, but none have been widely effective. PLP therapies vary from pharmacological options such as anesthetics (26), antidepressants (7, 26), and botulism toxin injections (7) to interventional treatments such as spinal cord stimulation (27), surgery (26), nerve block (26), neuromodulation (27), sensory discrimination (28), mental imagery (29), mirror therapy (26, 30), and virtual reality (VR) (12) treatments.

A number of these PLP therapies, including sensory discrimination, mental imagery, mirror therapy, and VR, attempt to normalize the cortical representation of the missing limb and improve the correspondence between actual and predicted sensory feedback. For instance, the use of anesthetic on the residual limb seems to be effective at reducing PLP when the injection induces a cortical reorganization (9). Sensory discrimination therapy uses tactile perception tasks presented at the residual limb to provide inputs from the amputated area and may reverse the cortical reorganization that is generating the pain (28, 31). The mirror box technique has also proven to be successful in reducing pain for some individuals (32, 33). In this intervention, a mirror is placed at the subject’s midline, and the subject watches the normal limb in the mirror while attempting to move both limbs in synchrony (34). Seeing the missing limb increases the individual’s sense of control of the PL and may reduce pain (6, 35). A limitation of the mirror box technique is the poor verisimilitude of the sensory feedback provided from the missing limb. The participant may have the visual illusion that the phantom extremity is moving, but the apparatus is crude and the illusion often not compelling. Patients cannot independently control the mirrored extremity, so only symmetric actions can be modeled.

Some of these limitations can be overcome using VR because this technology can provide visual input that is more varied and realistic than that provided by a mirror (36–38). Indeed, Ortiz-Catalan et al. (36) recently reported the experiences of a single subject with chronic upper-limb phantom pain who had failed mirror therapy. They employed a VR system to create an image of the missing hand on a computer monitor and used surface EMG data from the residual limb to enable the subject to control the hand and perform a series of reaching movements. The use of this system reduced the subject’s pain (36). Similar beneficial effects have also been obtained in larger samples of PLP patients (12, 37–39), reinforcing the potential utility of VR in PLP treatment. Mercier and Sirigu (38) reported an average pain reduction of 38% in eight individuals with upper-limb amputation who were trained to use the residual limb to match the movements of a virtual limb created from a mirror image of the intact limb. Similarly, Perry et al. (39) showed an average pain reduction of 40% in five upper-limb amputees who were trained with 20 sessions of active and passive imitation of an avatar’s movements. Using motion-tracking of the residual limb to create and control a virtual limb, Cole et al. (40) showed a beneficial effect after a single session of VR treatment in 10 of 14 individuals with PLP; furthermore, average pain reduction was 64%. These data suggest that VR systems that allow participants to directly control the virtual limb have significant potential to reduce PLP (40).

In the present study, we describe our preliminary findings in the treatment of PLP using a low-cost VR system that provides an immersive and responsive virtual representation of the intact and missing lower extremities that the user can control through natural motion of his or her intact and residual limbs. Two individuals who experienced PLP after leg amputation participated in a series of VR treatment sessions wherein they played custom games that require the use of both legs. The data suggest that this approach has substantial potential as a treatment for PLP.

## Materials and Methods

### Case Studies

Subject 1 was a late-middle-aged, hypertensive, diabetic person who underwent a right transtibial amputation for peripheral vascular disease 11 months before treatment. Subject 1 had a painful, non-healing foot wound for 6 months prior to amputation. After amputation, the pain persisted in the PL without change in character or severity. In the pretesting session, Subject 1 reported pain that varied in intensity from 2 to 10 and averaged 6 out of 10. All such ratings were gathered using a visual analog scale from 0 (no pain) to 10 (maximum level of pain). There were no factors that consistently altered the intensity of the pain. Subject 1 had tried numerous medication regimens without benefit. This participant could flex and extend his/her residual limb at the knee and did not experience telescoping of the PL. Subject 1 participated in only two sessions because of a newly diagnosed serious medical condition.

Subject 2 was a middle-aged person with peripheral vascular disease who underwent left transtibial amputation because of gangrene in the left foot. At the time of surgery, Subject 2 noted severe burning/aching pain in the left foot. That pain persisted in the PL that developed after the amputation. Subject 2 reported a clear sense of persistence of the lower leg and foot and felt that s/he could flex and extend the phantom foot but not wiggle its toes. After failing multiple therapies, including gabapentin, narcotics, tricyclics, and nerve blocks, Subject 2 was enrolled in our research project 7 months after the amputation. In the pretesting session, this participant reported a pain range from 4 to 10 out of 10, with an average of 7 out of 10. Subject 2 took part in four VR sessions over the course of approximately 6 weeks.

### Procedure

The format of each session was identical: after the VR apparatus was set up, the participant rated current pain on the same 0 to 10 scale and then trained with our VR system for approximately 1 h. The participants sat in their own wheelchair throughout the session. Treatment always started with at least 20 min of the most active game (*Quest for Fire*, described below), as it required vigorous use of the amputated limb. For the remaining time, the participant was free to choose which games to play. At the end of the hour, the participant was asked to rate the present severity of pain on the same 0 to 10 scale. To assess the design of the VR system, participants were asked to rate the *Quest for Fire* and *Chess* games on the System Usability Scale (41) after the final VR treatment.

All experimental procedures were approved by the University of Pennsylvania Institutional Review Board under protocol #823287. During recruitment, participants were told they could withdraw from the study at any point without providing an explanation and without any consequences. Enlisted participants gave informed consent and were compensated.

### VR Hardware and Software

**Figure 1:**
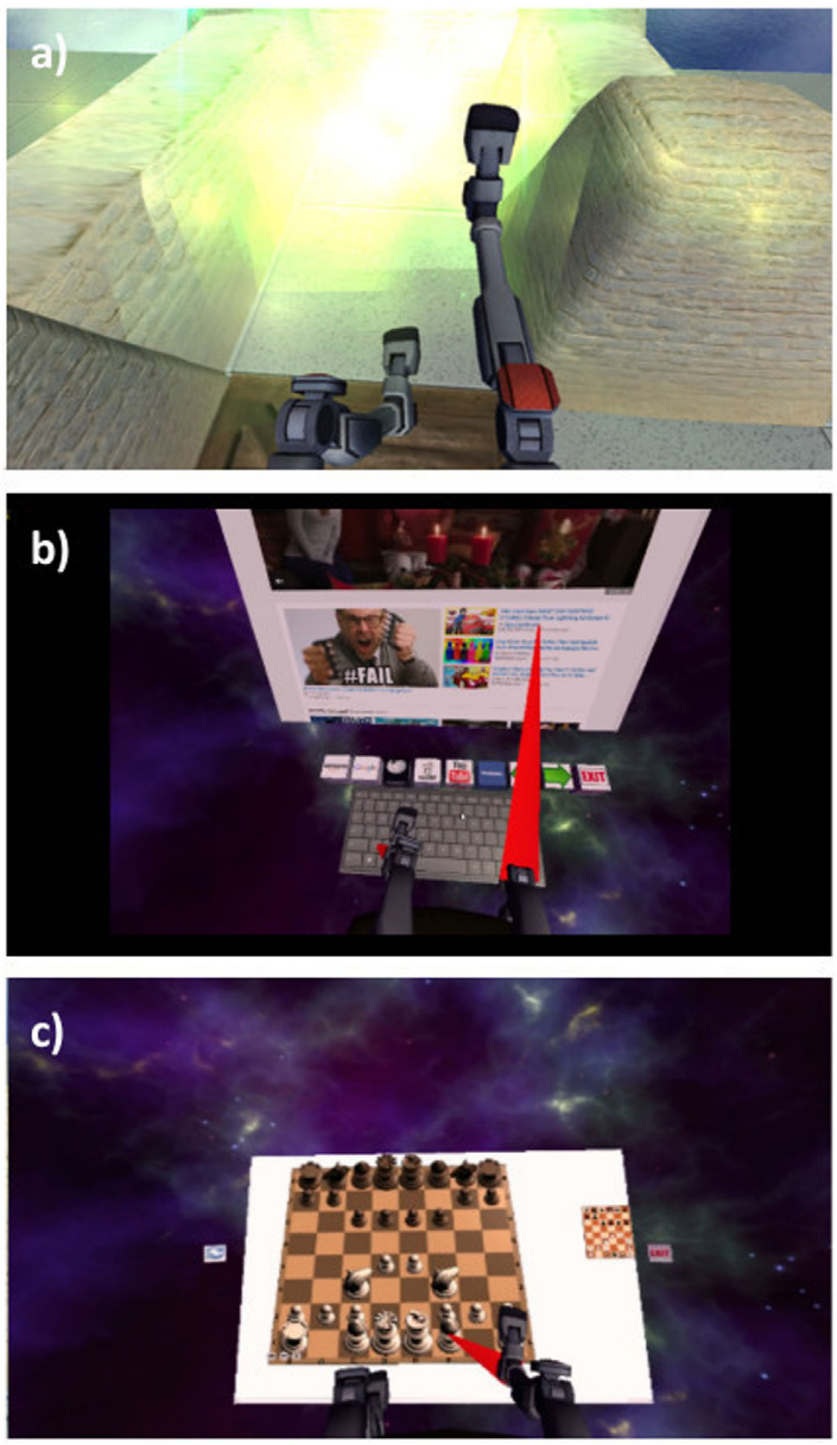
Participants’ view of the **(A)** *Quest for Fire*, **(B)** *Web browser*, and **(C)** *Chess* games. The *Checkers* game looks very similar to *Chess*.

**Figure 2:**
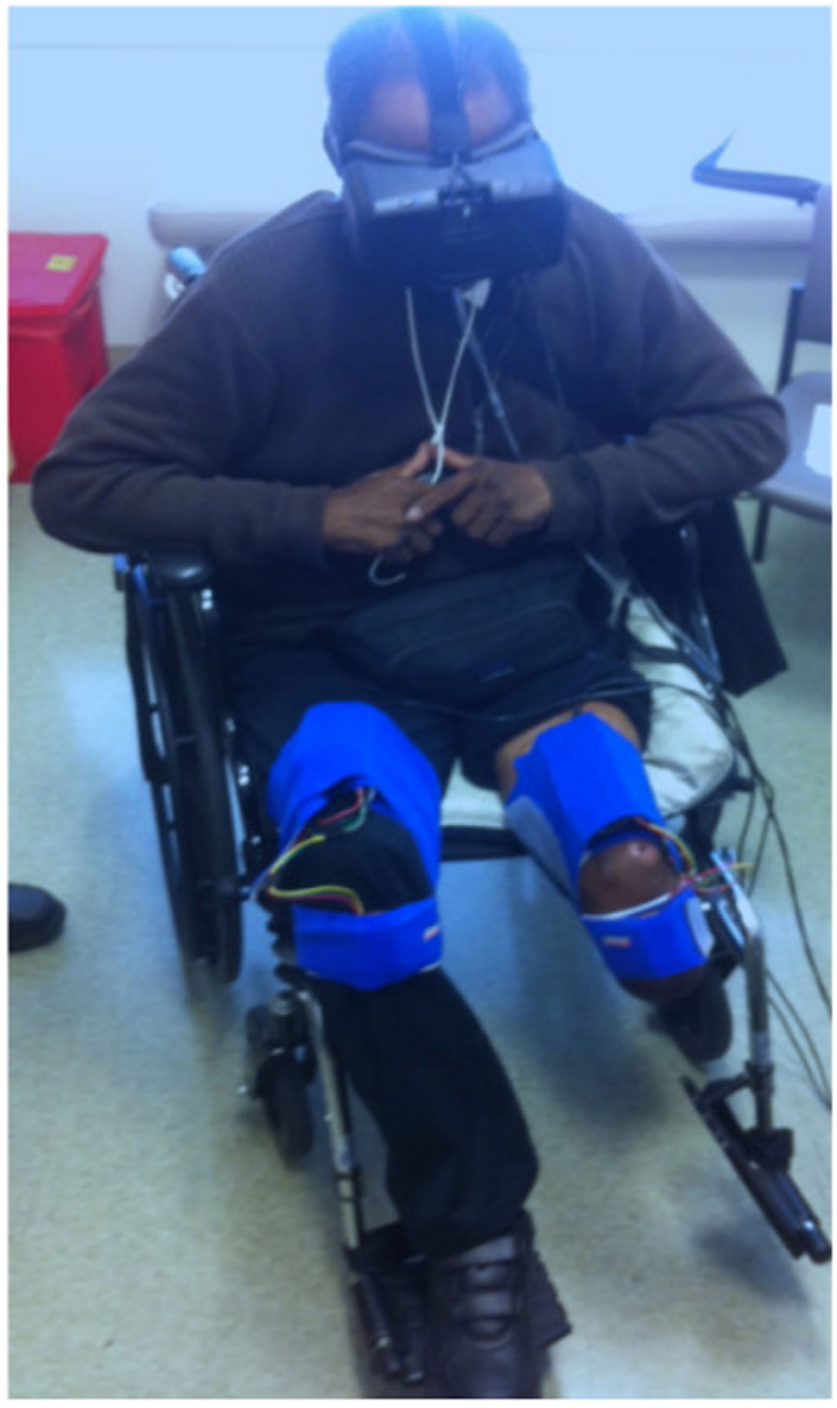
Subject 2 using the virtual reality system.

As our aim was to develop an affordable VR treatment for individuals who experience PLP, we used low-cost, high-quality components that are commercially available. First, the VR environment was presented using an Oculus Rift DK2 headset, a head-mounted display that provides three-dimensional graphical output. This headset adjusts the user’s view to match the orientation of his or her head in real time, providing an immersive and compelling view of the virtual environment. Second, we rigged a generic humanoid avatar (a robot) to allow the user to control the rotation of the hip and knee joints of both legs in a seated position. See Figure 1 for a screenshot of the user’s view in the *Quest for Fire* game. The avatar’s legs were controlled using four nine-degree-of-freedom inertial measurement units (IMUs) that were each mounted on a board and attached to the tops of the user’s thighs and the fronts of the anterior shins (directly below the knee joint) using stretchable fabric bands, as shown in Figure 2. To estimate the orientation of each of the four moving leg segments, Arduino microcontrollers were used to send readings from the IMUs to the computer, using a program written in the Arduino Programming language. A script written in Unity was then used to filter the readings from all four IMUs. The user could precisely control hip flexion/extension, hip adduction/abduction, and knee flexion/extension of each leg independently. Many events in each game caused sounds to help the user understand game contingencies and further increase the immersiveness of the system. These sounds were presented through the laptop speakers.

### Games

During the VR treatment, participants could play four games: *Quest for Fire, Web Browser, Chess*, and *Checkers* (see Figure 1). Loosely based on the Nintendo game Sokoban that was released in 1982 by Thinking Rabbit, *Quest for Fire* presents the player with a VR labyrinth environment. The avatar sits on a mobile chair and maneuvers around the virtual environment by moving their virtual legs (see Figure 1). The goal of each level is to reach the fiery portal at the end of the labyrinth by pushing crates into pits so that they no longer impede one’s path. This game has 17 levels that increase in complexity. Sounds effects were provided for crates sliding across the floor, crates falling into pits, the motion of the user’s chair, and the user entering a portal. In the *Web Browser* virtual environment, the user is presented with a virtual keyboard and a computer screen showing content from the Internet. Leg motions enable the user to navigate the Internet by moving the cursor and typing on a virtual keyboard. Click sounds were provided when participants clicked the VR keyboard or VR computer screen. In *Chess* and *Checkers*, the participant plays against a standard chess or checkers algorithm by identifying a piece to move using the virtual legs and then directing the virtual legs to the location to which he or she wants to move the piece. Click sounds were provided when participants clicked on a piece, along with sounds indicating the piece’s movement. Playing the games required the user to lift the legs by rotating at the hips, flex the knees, and execute different coordinated movements; therefore, participants were instructed to take breaks whenever they needed. Neither participant interrupted a session as a result of physical or mental fatigue.

## Results

**Figure 3:**
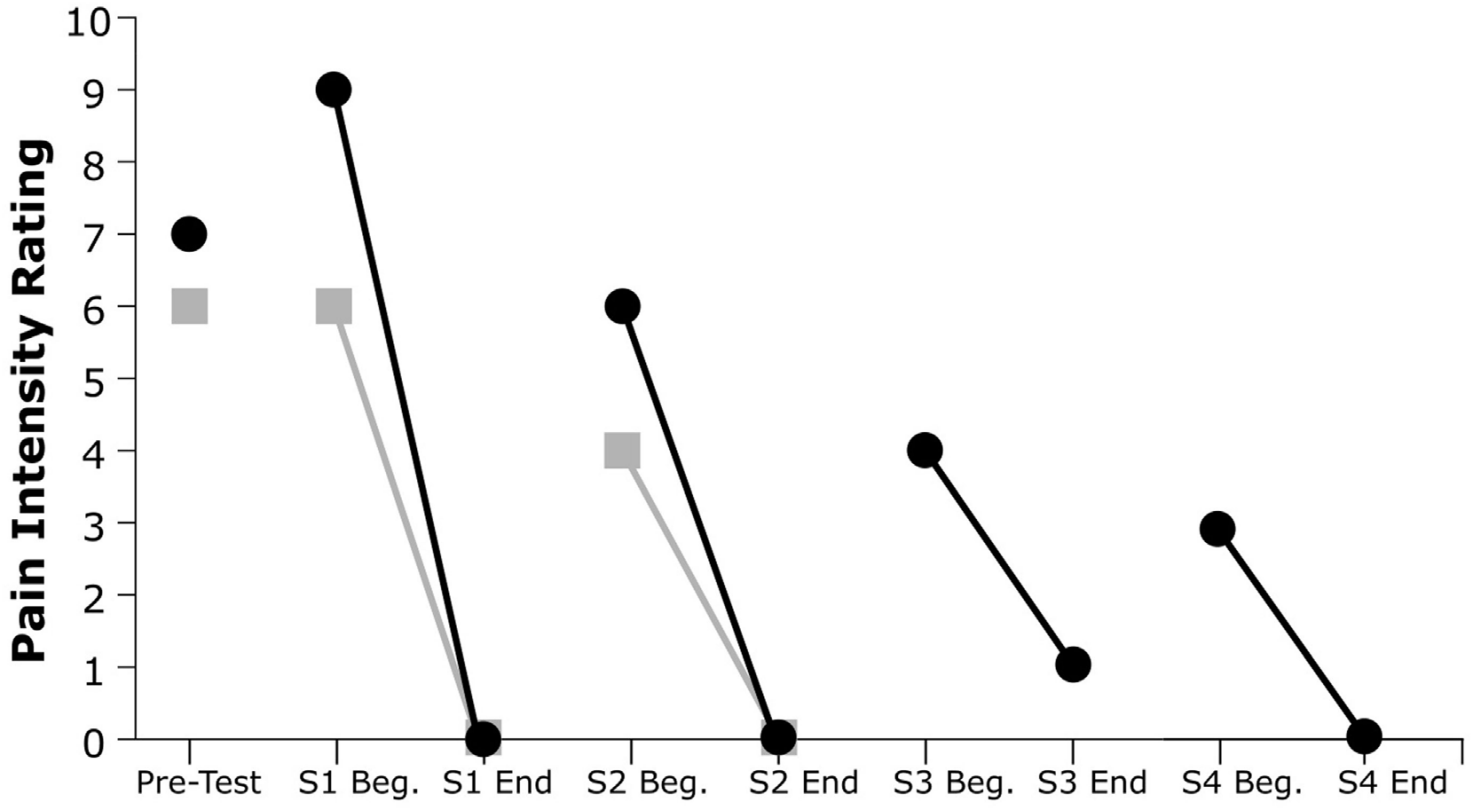
Pain intensity ratings from pretesting and at the beginning and end of each session (S). Gray squares indicate Subject 1’s ratings, and black circles indicate Subject 2’s ratings.

As shown in Figure 3, both subjects exhibited a substantial decline in pain immediately after each VR treatment session. Subject 1’s post-session (versus pre-session) pain intensity ratings diminished by 100% in both session 1 and session 2, while Subject 2’s post-session pain ratings diminished by an average of 93.7%. All but one of the six recorded post-session pain scores were at the minimum value of 0 out of 10, indicating no pain at all.

Furthermore, both participants showed a reduction in pretreatment pain severity in subsequent sessions and a progressive decrease of PLP across sessions. This trend was evident for both participants: Subject 1’s pain ratings decreased by 22% from the beginning of session 1 to the beginning of session 2, whereas Subject 2’s pain ratings showed a decrease of 67% from the beginning of session 1 to the beginning of session 4.

Qualitative feedback given during the experiment was also informative. Both subjects were highly enthusiastic about the system and were eager to continue the study, but they could not continue for health (Subject 1) and personal (Subject 2) reasons. Finally, it should be noted that Subject 2 reported that his overall level of activity improved dramatically over the course of the experiment. For example, after two sessions Subject 2 walked to the local grocery store using a lower-limb prosthesis for the first time.

Data from the System Usability Scale (41) demonstrated generally favorable ratings for usability of the system. Subject 2 scored the *Quest for Fire* and *Chess* games 70 and 83 out of 100, respectively; Subject 1 scored the same activities 40 and 78, respectively. Three of these four ratings are within the acceptable range (above 50 out of 100). Informal comments from Subject 1 indicated that the low rating for *Quest for Fire* reflected the frustration s/he encountered when learning to make the avatar move around the labyrinth.

Information regarding the sense of agency of the VR limb, the point during the session at which participants noted a reduction in PLP, and the possible association between level of fatigue and PLP was not obtained.

## Discussion

Preliminary data from the two participants suggests that our VR system may be a useful therapy for PLP. Indeed, both individuals reported a sizable decrease in PLP immediately after each 1-h-long VR session and a progressive reduction of pretest pain across sessions. As noted in the Section “Introduction,” prior work has demonstrated that VR may be of benefit in the treatment of PLP (38–40). Although the data must be interpreted with caution given the small sample size in our study, as well as in other investigations (36, 37), we note that the pain reduction achieved within a session was larger in our subjects than that reported in some previous studies (12, 40, 42), as both individuals were pain free after most VR sessions. Our subjects also did not report an increase in pain during the training, as had been observed in some previous research (38).

Although formal data are lacking, we believe that the variety and quality of the activities offered to the participants may have contributed to our promising results. Subject engagement may have been a limiting factor in the success of other VR systems developed to alleviate PLP, which in turn may be attributable to the repetitive and simple nature of the tasks implemented in some investigations. For example, Perry et al. (39) asked subject to pronate or supinate the wrist, and other investigators employed a simple reach and grasp task (40, 42–44) or press and release of a foot pedal (40). Other studies that have used more entertaining VR activities, like arranging a puzzle (45) or racing games (36, 46), have offered only a single game during the training. Our subjects were afforded a suite of games, were permitted to allocate most of their time according to their interests, and reported the tasks to be interesting and fun. Current research with our system is exploring the potential contributions of factors such as engagement, sense of agency, and level of effort to any observed treatment effects.

By using IMUs attached to the individual’s thighs and shins, our VR system allowed subjects to perform bilateral and unsynchronized leg movements, thereby providing subjects with the experience of being in full control of the virtual PL. This setup contrasts with many studies in which the visual image of the intact limb was transposed into the space of the phantom to create the virtual limb; such systems permit only bilateral synchronized movements [(38, 39, 45), but see Ref (40). for a counter-example]. As argued by Perry et al. (47), VR approaches that provide more lifelike feedback may be substantially more effective because they enable more diverse limb movements and provide richer sensory cues.

Importantly, our system uses the Oculus Rift headset to generate high-quality immersive VR. Many previous studies were carried out in non-immersive settings, with the virtual or augmented environment presented as a two-dimensional image on a computer monitor (36, 39, 46) or as a mirror reflection (38, 42, 44, 45). Although several recent studies have also employed immersive VR (36, 42–44), the environment presented in these studies was typically simple, such as a basic 3D world where a single unique object was presented. The rich virtual environments employed in our research may facilitate treatment benefit by increasing motivation and/or providing more lifelike visual cues.

Finally, our system is relatively easy to use. VR systems that employ myoelectric recording from the residual limb to create the VR limb (36, 46, 48) have used up to eight electrodes, which take time and skill to place. The use of simple inertial sensors represents a practical advantage and reduces the need for supervision; our system requires only a few minutes to set up and does not require technological expertise to operate. We believe it would be feasible to create a version that could be used at home without assistance, opening the door for a low-cost, convenient, effective PLP management strategy.

Although our investigation was not designed to explore the pathophysiology of PLP, we believe our data are in general agreement with the hypothesis that PLP is due to the incongruence or lack of correspondence between predicted and actual sensory and motor feedback regarding the extremity (5, 20). Following this line of reasoning, if loss of sensory feedback causes a degradation of sensory–motor representations relevant to the missing extremity, interventions that provide feedback relevant to the planned action of the missing extremity should reduce pain (15, 16).

A major limitation of our study is the small sample size. Still, it is encouraging that both participants responded strongly and reliably to our treatment. A further limitation of the present study is that our VR system provides visual and audio feedback, but not haptic (touch) feedback. As previous works suggest that haptic feedback increases the likelihood of improvement in PLP in some individuals (42–44), we intend to include haptic feedback in a future version of our system. An additional potential limitation is the fact that the avatar had robot-like rather than lifelike legs; although it is often assumed that “realism” enhances the effects of VR, it is noteworthy that our system achieved strong effects leg depictions that were responsive but not lifelike. A final limitation is that one of our subjects rated one game (*Quest for Fire*) as low in usability.

Our VR system continues to evolve; we have made several changes to the *Quest for Fire* software to improve its ease of use. Additionally, we have developed a version of the hardware that incorporates electromagnetic motion tracking rather than IMU-based tracking of leg position; this modification will address the fact that the IMU signals tended to drift during vigorous motion, contributing to participant frustration. We have also improved both visual and auditory feedback; for example, the new version of the system offers a more realistic reproduction of the limbs. Finally, we have upgraded the VR hardware with a new Oculus Rift that features built-in head position tracking and headphones, both of which increase the immersiveness of the VR environment. The upgraded system is currently being tested in a larger cohort of subjects who experience PLP.

To conclude, our VR system provided participants with an immersive VR experience while they played a variety of entertaining games using both legs. This system has shown clear potential for the treatment of PLP, achieving a substantial reduction in PLP in two individuals over only two to four sessions. Because of its low cost and ease of use, this system is a potential prototype for home-based treatment of PLP. Finally, the positive results in the treatment of PLP reported here and in previous studies support the view that VR may be a useful treatment for different forms of chronic pain or other acquired brain disorders, such as stroke (49) or spinal cord injury (50).

## Author Contributions

EA wrote a first draft of the manuscript and conducted the testing sessions. AM designed and implemented the VR system. KK designed the study and the VR system, and revised the manuscript. LB designed the study and revised the manuscript. HC designed the study, conducted the testing sessions and revised the manuscript.

## Conflict of Interest Statement

The authors declare that the research was conducted in the absence of any commercial or financial relationships that could be construed as a potential conflict of interest.

## References

[1] Weeks SR Anderson-Barnes VC Tsao JW. Phantom limb pain: theories and therapies. Neurologist (2010) 16:277–86. 10.1097/NRL.0b013e3181edf128 20827116

[2] Ehde DM Jensen MP Engel JM Turner JA Hoffman AJ Cardenas DD. Chronic pain secondary to disability: a review. Clin J Pain (2003) 19(1):3–17. 10.1097/00002508-200301000-00002 12514452

[3] Kooijman CM Dijkstra PU Geertzen JH Elzinga A van der Schans CP. Phantom pain and phantom sensations in upper limb amputees: an epidemiological study. Pain (2000) 87:33–41. 10.1016/S0304-3959(00)00264-5 10863043

[4] Giummarra MJ Gibson SJ Georgiou-Karistianis N Bradshaw JL. Central mechanisms in phantom limb perception: the past, present and future. Brain Res Rev (2007) 54(1):219–32. 10.1016/j.brainresrev.2007.01.009 17500095

[5] Flor H Nikolajsen L Jensen TS. Phantom limb pain: a case of maladaptive CNS plasticity? Nat Rev Neurosci (2006) 7(11):873–81. 10.1038/nrn1991 17053811

[6] Ramachandran VS Altschuler EL. The use of visual feedback, in particular mirror visual feedback, in restoring brain function. Brain (2009) 132(7):1693–710. 10.1093/brain/awp135 19506071

[7] McCormick Z Chang-Chien G Marshall B Huang M Harden RN. Phantom limb pain: a systematic neuroanatomical-based review of pharmacologic treatment. Pain Med (2014) 15(2):292–305. 10.1111/pme.12283 24224475

[8] Nyström B Hagbarth KE. Microelectrode recordings from transected nerves in amputees with phantom limb pain. Neurosci Lett (1981) 27(2):211–6. 10.1016/0304-3940(81)90270-6 7322453

[9] Birbaumer N Lutzenberger W Montoya P Larbig W Unertl K Töpfner S Effects of regional anesthesia on phantom limb pain are mirrored in changes in cortical reorganization. J Neurosci (1997) 17(14):5503–8. 9204932 10.1523/JNEUROSCI.17-14-05503.1997 PMC6793813

[10] Melzack R Israel R Lacroix R Schultz G. Phantom limbs in people with congenital limb deficiency or amputation in early childhood. Brain (1997) 120(9):1603–20. 10.1093/brain/120.9.1603 9313643

[11] Brugger P Kollias SS Müri RM Crelier G Hepp-Reymond MC Regard M. Beyond re-membering: phantom sensations of congenitally absent limbs. Proc Natl Acad Sci U S A (2000) 97(11):6167–72. 10.1073/pnas.100510697 10801982 PMC18576

[12] Dunn J Yeo E Moghaddampour P Chau B Humbert S Virtual and augmented reality in the treatment of phantom limb pain: a literature review. NeuroRehabilitation (2017) 40(4):595–601. 10.3233/NRE-171447 28211829

[13] Merzenich MM Nelson RJ Stryker MP Cynader MS Schoppmann A Zook JM. Somatosensory cortical map changes following digit amputation in adult monkeys. J Comp Neurol (1984) 224(4):591–605. 10.1002/cne.902240408 6725633

[14] Ramachandran VS Rogers-Ramachandran D Phantom limbs and neural plasticity. Arch Neurol (2000) 57(3):317–20. 10.1001/archneur.57.3.317 10714655

[15] Karl A Birbaumer N Lutzenberger W Cohen LG Flor H. Reorganization of motor and somatosensory cortex in upper extremity amputees with phantom limb pain. J Neurosci (2001) 21(10):3609–18. 11331390 10.1523/JNEUROSCI.21-10-03609.2001 PMC6762494

[16] Karl A Mühlnickel W Kurth R Flor H. Neuroelectric source imaging of steady-state movement-related cortical potentials in human upper extremity amputees with and without phantom limb pain. Pain (2004) 110(1):90–102. 10.1016/j.pain.2004.03.013 15275756

[17] Flor H Elbert T Knecht S Wienbruch C Pantev C Birbaumer N Phantom-limb pain as a perceptual correlate of cortical reorganization following arm amputation. Nature (1995) 375(6531):482–184. 10.1038/375482a0 7777055

[18] Flor H Elbert T Mühlnickel W Pantev C Wienbruch C Taub E. Cortical reorganization and phantom phenomena in congenital and traumatic upper-extremity amputees. Exp Brain Res (1998) 119(2):205–12. 10.1007/s002210050334 9535570

[19] Nikolajsen L Ilkjaer S Krøner K Christensen JH Jensen TS. The influence of preamputation pain on postamputation stump and phantom pain. Pain (1997) 72(3):393–405. 10.1016/S0304-3959(97)00061-4 9313280

[20] Harris AJ Cortical origin of pathological pain. Lancet (1999) 354(9188):1464–6. 10.1016/S0140-6736(99)05003-5 10543687

[21] da Paz AC Jr. Braga LW Downs JR III. A preliminary functional brain study on amputees. Appl Neuropsychol (2000) 7(3):121–5. 10.1207/S15324826AN0703_1 11213758

[22] Reilly KT Mercier C Schieber MH Sirigu A. Persistent hand motor commands in the amputees’ brain. Brain (2006) 129(8):2211–23. 10.1093/brain/awl154 16799174

[23] Flor H Diers M Andoh J. The neural basis of phantom limb pain. Trends Cogn Sci (2013) 17(7):307–8. 10.1016/j.tics.2013.04.007 23608362

[24] Makin TR Scholz J Filippini N Slater DH Tracey I Johansen-Berg H. Phantom pain is associated with preserved structure and function in the former hand area. Nat Commun (2013) 4:1570. 10.1038/ncomms2571 23463013 PMC3615341

[25] Kikkert S Kolasinski J Jbabdi S Tracey I Beckmann CF Johansen-Berg H Revealing the neural fingerprints of a missing hand. Elife (2016) 5:e15292. 10.7554/eLife.15292 27552053 PMC5040556

[26] Richardson C Kulkarni J. A review of the management of phantom limb pain: challenges and solutions. J Pain Res (2017) 10:1861–70. 10.2147/JPR.S124664 28860841 PMC5558877

[27] Aiyer R Barkin RL Bhatia A Gungor S. A systematic review on the treatment of phantom limb pain with spinal cord stimulation. Pain (2017) 7(1):59–69. 10.2217/pmt-2016-0041 27780402

[28] Flor H Denke C Schaefer M Grüsser S. Effect of sensory discrimination training on cortical reorganisation and phantom limb pain. Lancet (2001) 357(9270):1763–4. 10.1016/S0140-6736(00)04890-X 11403816

[29] MacIver K Lloyd DM Kelly S Roberts N Nurmikko T. Phantom limb pain, cortical reorganization and the therapeutic effect of mental imagery. Brain (2008) 131(8):2181–91. 10.1093/brain/awn124 18567624 PMC2494616

[30] Thieme H Morkisch N Rietz C Dohle C Borgetto B The efficacy of movement representation techniques for treatment of limb pain—a systematic review and meta-analysis. J Pain (2016) 17(2):167–80. 10.1016/j.jpain.2015.10.015 26552501

[31] Moseley GL Zalucki NM Wiech K Tactile discrimination, but not tactile stimulation alone, reduces chronic limb pain. Pain (2008) 137(3):600–8. 10.1016/j.pain.2007.10.021 18054437

[32] Chan BL Witt R Charrow AP Magee A Howard R Pasquina PF Mirror therapy for phantom limb pain. N Engl J Med (2007) 357(21):2206–7. 10.1056/NEJMc071927 18032777

[33] Hasanzadeh Kiabi F Habibi MR Soleimani A Emami Zeydi A Mirror therapy as an alternative treatment for phantom limb pain: a short literature review. Korean J Pain (2013) 26(3):309–11. 10.3344/kjp.2013.26.3.309 23862009 PMC3710949

[34] Ramachandran VS Rogers-Ramachandran D. Synaesthesia in phantom limbs induced with mirrors. Proc R Soc Lond B Biol Sci (1996) 263(1369):377–86. 10.1098/rspb.1996.0058 8637922

[35] MacLachlan M McDonald D Waloch J. Mirror treatment of lower limb phantom pain: a case study. Disabil Rehabil (2004) 26(14–15):901–4. 10.1080/09638280410001708913 15497919

[36] Ortiz-Catalan M Sander N Kristoffersen MB Håkansson B Brånemark R. Treatment of phantom limb pain (PLP) based on augmented reality and gaming controlled by myoelectric pattern recognition: a case study of a chronic PLP patient. Front Neurosci (2014) 8:24. 10.3389/fnins.2014.00024 24616655 PMC3935120

[37] Murray CD Pettifer S Howard T Patchick EL Caillette F Kulkarni J The treatment of phantom limb pain using immersive virtual reality: three case studies. Disabil Rehabil (2007) 29(18):1465–9. 10.1080/09638280601107385 17729094

[38] Mercier C Sirigu A. Training with virtual visual feedback to alleviate phantom limb pain. Neurorehabil Neural Repair (2009) 23(6):587–94. 10.1177/1545968308328717 19171946

[39] Perry BN Alphonso AL Tsao JW Pasquina PF Armiger RS Moran CW A virtual integrated environment for phantom limb pain treatment and modular prosthetic limb training. Virtual Rehabilitation (ICVR), 2013 International Conference on IEEE Philadelphia (2013). p. 153–7.

[40] Cole J Crowle S Austwick G Henderson Slater D. Exploratory findings with virtual reality for phantom limb pain; from stump motion to agency and analgesia. Disabil Rehabil (2009) 31:846–54. 10.1080/09638280802355197 19191061

[41] Brooke J SUS-A quick and dirty usability scale. Usability Eval Industry (1996) 189(194):4–7.

[42] Wake N Sano Y Oya R Sumitani M Kumagaya SI Kuniyoshi Y Multimodal virtual reality platform for the rehabilitation of phantom limb pain. Conference on Neural Engineering (NER), 2015 7th International IEEE/EMBS Montpellier (2015). p. 787–90.

[43] Ichinose A Sano Y Osumi M Sumitani M Kumagaya SI Kuniyoshi Y. Somatosensory feedback to the cheek during virtual visual feedback therapy enhances pain alleviation for phantom arms. Neurorehabil Neural Repair (2017) 31(8):717–25. 10.1177/1545968317718268 28691602

[44] Sano Y Wake N Ichinose A Osumi M Oya R Sumitani M Tactile feedback for relief of deafferentation pain using virtual reality system: a pilot study. J Neuroeng Rehabil (2016) 13(1):61. 10.1186/s12984-016-0161-6 27353194 PMC4924286

[45] Murray CD Patchick E Pettifer S Howard T Caillette F Kulkarni J Investigating the efficacy of a virtual mirror box in treating phantom limb pain in a sample of chronic sufferers. Int J Disabil Hum Dev (2006) 5(3):227–34. 10.1515/IJDHD.2006.5.3.227

[46] Ortiz-Catalan M Guðmundsdóttir RA Kristoffersen MB Zepeda-Echavarria A Caine-Winterberger K Kulbacka-Ortiz K Phantom motor execution facilitated by machine learning and augmented reality as treatment for phantom limb pain: a single group, clinical trial in patients with chronic intractable phantom limb pain. Lancet (2016) 388(10062):2885–94. 10.1016/S0140-6736(16)31598-7 27916234

[47] Perry BN Mercier C Pettifer SR Cole J Tsao JW Virtual reality therapies for phantom limb pain. Eur J Pain Lond Engl (2014) 18:897–9. 10.1002/ejp.559 25045000

[48] Lendaro E Mastinu E Håkansson B Ortiz-Catalan M. Real-time classification of non-weight bearing lower-limb movements using EMG to facilitate phantom motor execution: engineering and case study application on phantom limb pain. Front Neurol (2017) 8:470. 10.3389/fneur.2017.00470 28955294 PMC5601955

[49] Hatem SM Saussez G della Faille M Prist V Zhang X Dispa D Rehabilitation of motor function after stroke: a multiple systematic review focused on techniques to stimulate upper extremity recovery. Front Hum Neurosci (2016) 10:442. 10.3389/fnhum.2016.00442 27679565 PMC5020059

[50] Pozeg P Palluel E Ronchi R Solcà M Al-Khodairy AW Jordan X Virtual reality improves embodiment and neuropathic pain caused by spinal cord injury. Neurology (2017) 89(18):1894–903. 10.1212/WNL.0000000000004585 28986411 PMC5664293

